# EDTA‐dependent pseudo thrombocytopenia mimicking dengue fever‐associated persistent thrombocytopenia: A case report

**DOI:** 10.1002/ccr3.4999

**Published:** 2021-10-23

**Authors:** Gentle Sunder Shrestha, Babin Basnet, Gaurav Nepal, Rajan Lamichhane, Prabin Gaire, Rayana Shrestha, Sabin Thapalia

**Affiliations:** ^1^ Department of Anaesthesiology Tribhuvan University Institute of Medicine Maharajgunj Nepal; ^2^ Bhimphedi Primary Health Care Center Bhimphedi Nepal; ^3^ Rani Primary Health Care Center Biratnagar Nepal; ^4^ Department of Internal Medicine Tribhuvan University Institute of Medicine Maharajgunj Nepal; ^5^ Department of Pathology Tribhuvan University Institute of Medicine Maharajgunj Nepal

**Keywords:** dengue, EDTA, platelet clumping, pseudothrombocytopenia, thrombocytopenia

## Abstract

No hemorrhagic manifestations, presence of platelet clumps on the peripheral blood smear, normal manual count, and normal autoanalyzer count after collecting blood in citrate vial help confirm the diagnosis of EDTA‐dependent thrombocytopenia.

## Graphical abstract

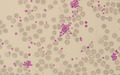

## 1 INTRODUCTION

There has been a dramatic improvement in cellular hematology after the emergence of hematology analyzers. Hematology analyzers utilize various technologies like electrical impedance, flow cytometry, and fluorescent flow cytometry to recognize cell types in a blood sample and to count them individually to generate a complete blood count with differential count. They have produced quick and accurate results most of the time.^1^, ^2^ Measurement of platelet counts using automated hematology analyzers is usually quite precise and accurate. However, the accuracy of automated platelet counts can be compromised when platelet clumping occurs in blood samples. Pseudothrombocytopenia or spurious thrombocytopenia, a relatively uncommon phenomenon, occurs in vitro when blood is collected in anticoagulant containing vial which leads to clumping of platelets. In such cases, the automatic hematology analyzer can not provide a precise count.^2^, ^3^ Spuriously low platelet counts are seen in ethylenediaminetetraacetic acid (EDTA)‐anticoagulated blood; however, other anticoagulants such as citrate, heparin, and oxalate have also been reported.^2^ Furthermore, platelet satellitism, platelet cold agglutinins, large platelets, and improper blood withdrawal techniques are also associated with pseudo thrombocytopenia.^1^, ^4^ In this case report, we report a case of EDTA‐dependent pseudo thrombocytopenia in a 17 years old female diagnosed with dengue fever with warning signs. She was initially misdiagnosed as dengue‐associated persistent thrombocytopenia. However, blood was later collected in a non‐EDTA vial and sent for analysis. Both automated and manual counting were performed, revealing normal platelet count.

## 2 CASE PRESENTATION

A 17‐year‐old female presented to the emergency of Tribhuvan University Teaching Hospital (TUTH) with chief complaints of a week‐long fever and cough. The fever was on and off, associated with chills and rigor, sweating, retro‐orbital pain, and diffuse headache. There was no diurnal variation, abdominal pain, vomiting, and diarrhea. There were no history of photophobia and loss of consciousness. Neck rigidity and delirium were absent. Her cough was dry, non‐productive, and without hemoptysis. It was associated with bilateral diffuse chest pain, and shortness of breath (SOB), worsened with exertion. She denies any contact with a tuberculosis patient. She denies any rash, easy bruising, joint pain, or bone pain. Her past medical and surgical history was unremarkable. She was a nonsmoker and did not consume alcohol. She was not under any mediation and did not report any drug allergies.

On presentation she was well oriented to time, place, and person, her GCS was 15, pulse was 140 beats per minute, blood pressure (BP) was 120/90 mmHg, respiratory rate was 20 breath/min, oxygen saturation was 84% at room air, and she was febrile with a temperature of 102°F. Her tongue and mucosa were dry. There was no lymphadenopathy, pedal edema, pallor, icterus, and cyanosis. On chest examination, there was a dull note on percussion bilaterally on the inframammary and infrascapular region. There was decreased air entry on both sides and decreased vocal resonance on auscultation. On cardiac examination, she had normal heart sounds and without murmurs. There was mild abdominal tenderness without organomegaly. The examination of other organ systems was unremarkable.

Empiric antibiotics, IV fluids, antipyretics, and supplemental oxygen were administered. Her total leucocyte count was 8600/mm^3^, hemoglobin was 12.5 gm%, PCV was 36.9%, RBC was 4 million/mm^3^, and platelet was 15,000/ mm^3^. Her renal function test results were within normal limits. Total bilirubin was 7 µMol/L, direct bilirubin was 2 µMol/L, and AST and ALT were 80 and 130 U/L, respectively, alkaline phosphatase was 590 U/L, albumin was 29 g/L. Urine microscopy and routine examination were normal.

RT‐PCR test for COVID‐19 was negative. Chest X‐ray showed bilateral pleural effusion, USG of abdomen, and pelvis showed hepatomegaly and fluid in the pouch of Douglas. Induced sputum on Gram and AFB staining showed no organism. Malarial parasites could not be seen on blood smears. Serology of brucella, leptospirosis and scrub typhus, HIV, HBV, HCV, and HAV were negative. However, she tested positive for dengue NS1 antigen.

With features like abdominal tenderness, hepatomegaly, features suggestive of fluid accumulation, and thrombocytopenia, she was diagnosed with dengue fever with warning signs. She was treated in ICU. Her vitals were monitored continuously. Daily fluid intake and output were recorded. Complete blood counts and renal function tests were sent frequently. She was managed symptomatically.

Her platelet counts were 15,000/mm^3^, 6000/mm^3^, and 15,000/mm^3^ on days 1, 2, 3 of admission to ICU. She received 2 pints of platelet concentrate and 4 pints of platelet‐rich plasma on day 3 of admission in ICU. On the 4th day, her platelet counts were 8000/mm^3^ and on repeat testing 12 h later it was 25,000/mm^3^. On the 5th day, the platelet count was 10,000/mm^3^. She was again transfused with 2 pints of platelet‐rich plasma. The blood count was repeated after 12 h showing a platelet count of 25,000/mm^3^. Despite receiving multiple transfusions, the platelet count was not improving as expected.

**FIGURE 1:**
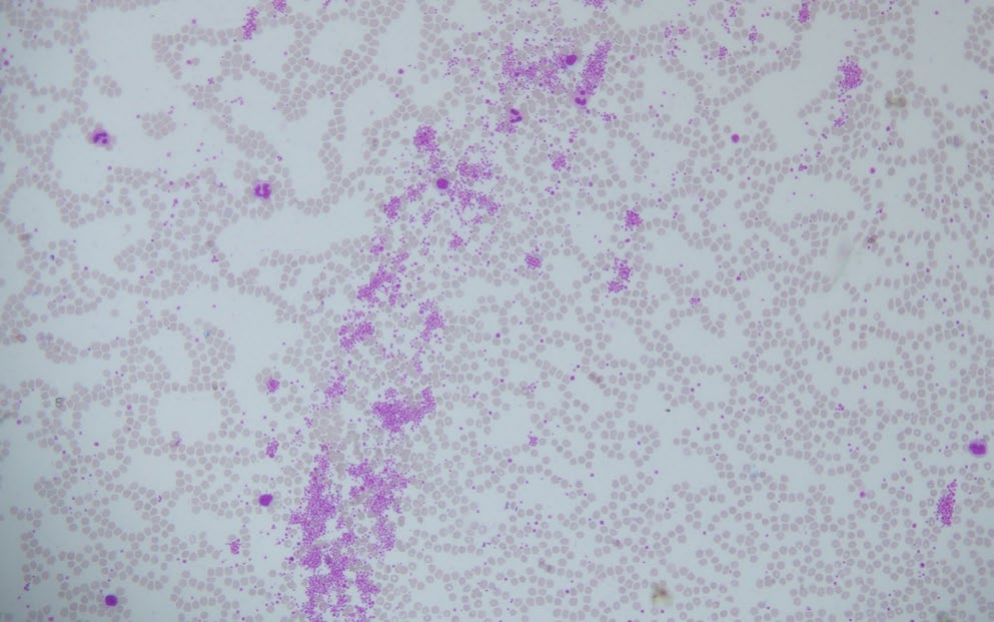
Peripheral smear of the ethylenediaminetetraacetic acid‐anticoagulated blood showing platelet clumping (200× zoom)

**FIGURE 2:**
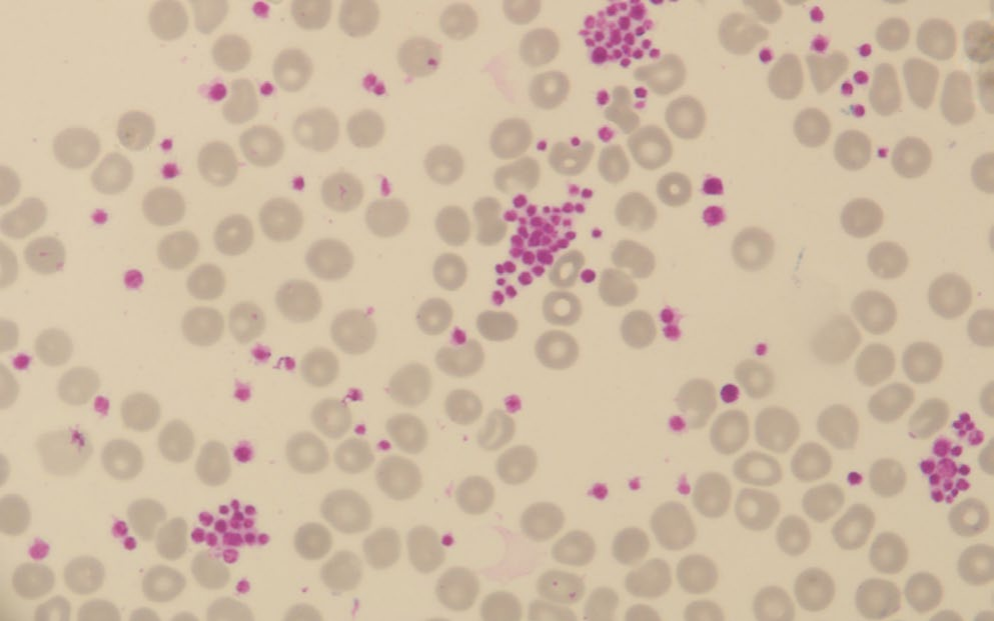
Peripheral smear of the ethylenediaminetetraacetic acid‐anticoagulated blood showing platelet clumping (1000× zoom)

Coombs test, antiplatelet antibody, and ANA (anti‐nuclear antibody) test were negative. PT/INR and PTT were normal. On the 6th day of admission, her platelets counts were 11,000/mm^3^. She received 2 pints of platelet‐rich plasma on that day. A peripheral blood smear (PBS) was sent, and a hematology consultation was done. Her PBS showed platelet clumping (Figures 1,2).

With the suspicion of pseudo thrombocytopenia, the blood sample was sent in the citrate vial, which showed a platelet count of 323,000/mm^3^. The next day, hemogram was performed in both the EDTA and the citrate vials, along with manual counting of the platelet. The report of the EDTA vial sample showed a platelet count of 8000/mm^3^, the platelet count of the citrate vial was 359,000/mm^3^, and the manual count was 335,000/mm^3^. Finally, she was observed in the general ward for 2 days and was subsequently discharged.

## 3 DISCUSSION

Thrombocytopenia is a common manifestation of dengue fever, and patients may have varying degrees of thrombocytopenia. Low platelets usually last for around 5 days after which platelet recovery begins and normalizes after about 10 days of fever.^5^ This usual course of the disease was not observed in our patient and her platelet count was not improving despite improvement in clinical status and receiving multiple platelet transfusions. Persistent thrombocytopenia following dengue shock syndrome has been reported in the literature.^6^, ^7^Initially, our patient was suspected to have the same and was treated with multiple platelet transfusions. However, persistent unresponsiveness and no evidence of bleeding and bleeding‐related complications ushered us to look for an alternative diagnosis.

Our patient's platelet antibody test was normal, and the coombs test was also negative, which effectively ruled out autoimmune‐mediated platelet destruction. Her hemogram shows isolated thrombocytopenia, which is uncommon in bone marrow suppression. Acute leukemia could have been suspected in our case, but isolated thrombocytopenia, normal PBS findings (normal leukocyte count and absence of blasts and atypical cells) were not in favor of leukemia. However, for confirmation, a bone marrow biopsy is required which was not performed. Isolated thrombocytopenia, normal indirect bilirubin level, normal urine microscopy, and biochemistry finding, normal renal function test, and coagulation profile further ruled out thrombotic thrombocytopenic purpura. The absence of joint pain, oral ulcers, a negative test for ANA, and the absence of typical rashes indicated that SLE was less likely. Immune thrombocytopenic purpura (ITP) could have been considered in this scenario but it required a bone marrow biopsy to confirm the diagnosis. However, it was withheld as the PBS showed the presence of platelet clumping, and citrate vial hemogram and manual counting showed normal platelet count. This made it clear that thrombocytopenia was false and was EDTA‐dependent.

First reported in 1973 by Shreiner and Bell,^8^the exact mechanism of EDTA‐dependent pseudo thrombocytopenia is unknown but it is proposed that the presence of EDTA‐dependent autoantibodies in plasma causes in vitro aggregation of platelets. The low temperature and effect of EDTA on calcium ions destabilize the platelet membrane glycoprotein complex IIb/IIIa. Glycoprotein IIb is observed to exist as a calcium‐dependent heterodimer which is complexed with glycoprotein IIIa. The epitope of glycoprotein IIb is normally hidden in the glycoprotein complex IIb/IIIa. When the calcium concentration is lowered dissociation of the dimer is seen, and the epitope of glycoprotein IIb is revealed. If EDTA‐dependent antiplatelet autoantibodies are present in plasma against these epitopes, autoantibodies bind to the epitopes and hence cause the aggregation of platelets.^2^, ^3^, ^4^, ^9^

Therefore, clinicians should pay attention to this easily diagnosed phenomenon. If unaware, it may lead to misdiagnosis and faulty treatment. Besides, this will increase the financial burden on patients due to unnecessary and expensive investigations. Some of them may undergo unnecessary intervention like bone marrow biopsy and even splenectomy if a physician suspects it as ITP. Though platelet‐related transfusion reactions are minimal as compared to red cells, some of the complications like transfusion‐related acute lung injury and transfusion‐associated circulatory overload are life‐threatening.^10^Therefore, in the case of persistent thrombocytopenia, PBS should be considered. If PBS shows evidence of platelet clumping, the sample should be collected in a non‐EDTA vial. Besides, manual counting of platelets by microscopy is helpful in such cases.

## 4 CONCLUSION

In any patient with dengue fever with thrombocytopenia not responding to platelets transfusion, EDTA‐dependent pseudo thrombocytopenia should be suspected. The diagnosis can be easily confirmed by ordering a peripheral blood smear with manual platelet counting or repeating a hemogram using a citrate‐buffered blood sample. These simple investigations can avoid unnecessary investigations, treatments, and complications.

## CONFLICT OF INTEREST

The authors declare no conflict of interest.

## AUTHOR CONTRIBUTIONS

GSS and GN contributed to writing the manuscript, its concept, the collection of case information, and manuscript revision. BB contributed to writing the manuscript and collection of data. RL and PG contributed to the literature review and interpretation of clinical findings. ST, GSS, RS, and GN were involved in the patient care team and contributed to the collection of case information. All authors approved the final version.

## 5 CONSENT

Written informed consent was obtained from the patient for publication of this case report and any accompanying images. A copy of the written consent is available for review by the Editor‐in‐Chief of this journal.

## Data Availability

The figure used to support the findings of this study are included within the article.
